# Characteristics of 3D-Integrated GaN Power Module Under Multi Heat Source Coupling

**DOI:** 10.3390/ma18051082

**Published:** 2025-02-28

**Authors:** Yijun Shi, Mingen Lv, Guoguang Lu, Caixing Hui, Liang He, Xinghuan Chen, Yuan Chen, Xiangjun Lu

**Affiliations:** 1 China Electronic Product Reliability and Environmental Testing Research Institute, Guangzhou 510610, China; syj20094870@sina.com (Y.S.); mingenlv@hnu.edu.cn (M.L.); he_liang_mail@163.com (L.H.); xhchen01@outlook.com (X.C.); chenyuan@ceprei.com (Y.C.); 2 School of Material Science and Engineering, Xiamen University of Technology, Xiamen 361024, China; hcx971022@163.com; 3 College of Electrical and Information Engineering, Hunan University, Changsha 410012, China

**Keywords:** 3D integration, GaN power module, multi-heat source coupling, threshold voltage

## Abstract

3D-integrated GaN power modules can effectively reduce parasitic parameters and enhance the power system’s performance. However, the heat from each power chip during operation can lead to a mutual thermal coupling effect, potentially causing performance drift of the GaN power chips. This work investigates the impact of the thermal coupling effect in a 3D-integrated GaN power module on the characteristics of its GaN power chips. The GaN power chips’ characteristics are measured before and after the other power chips in the 3D-integrated GaN power module and after applying *V*_GS_/*V*_DS_ = 3 V/1 V for 60 s. The results indicate that the thermal coupling effect in 3D-integrated GaN power modules can cause a rightward shift in the threshold voltage, reduce the response speed and on-state current, and also increase the leakage current of GaN power chips. In severe cases, the threshold voltage drift can reach up to 0.26 V, the device’s response time can increase by as much as 217 μs, the on-state current can decrease by 1.7 A, and the off-state leakage current can increase by more than 80 times. The impact of the thermal coupling effect is related to the direction of heat flow and the distance between chips. The closer the chips are to each other, the stronger the thermal coupling. It has a greater impact on the performance of chips near the bottom substrate and a lesser impact on the performance of chips at the top of the module. Typically, the influence of the thermal field generated by two chips working simultaneously is more significant than that of the thermal field generated by a single chip working alone.

## 1. Introduction

Wide bandgap semiconductor materials are highly suitable for various components of power systems, such as SnO_2_ or ZnO for energy storage elements, and gallium nitride (GaN) for power processing elements [1,2,3]. GaN exhibits characteristics such as high breakdown electric field strength, high electron mobility, high saturation electron drift velocity, high thermal conductivity, high thermal stability, low dielectric constant, strong radiation resistance, and high electron density from the strong polarization effect of GaN-based heterojunction [4,5]. These properties make GaN heterojunction an ideal semiconductor material for high-voltage, high-frequency, high-temperature, and high-power-density applications. The GaN high-electron-mobility transistor (HEMT) is a widely studied structure due to its technological maturity, simple fabrication process, and excellent performance [6,7,8,9]. It meets the requirements of next-generation power module systems for higher power, higher frequency, smaller size, and harsher operating environments.

To further reduce parasitic parameters in power module systems based on GaN power devices and enhance their operating frequency, efficiency, power density, and temperature characteristics [10], current approaches often involve integrating one or multiple GaN power devices with their respective driver circuits, ESD protection circuits, and over-current protection circuits onto a single chip (system-on-chip integration, SoC) [11,12,13,14,15,16], or packaging them within the same casing (system-in-package integration, SiP) [17,18,19,20,21,22,23,24]. SiP integration can be further categorized into planar packaging integration and three-dimensional stacked packaging integration (3D-integrated GaN power module) [25,26,27,28]. The 3D-integrated GaN power module involves integrating multiple GaN power chips and other control chips on a substrate through three-dimensional packaging and interconnection, creating a miniaturized device that provides system or subsystem functionality. This approach boasts high performance, high integration, and miniaturization, effectively reducing parasitic parameters in power module systems and improving their operating frequency, efficiency, and power density. Nowadays, the development of 3D-integrated GaN power modules has become one of the key research directions in GaN power devices.

As the size of the system decreases and operating frequency and power density increase, the electro-thermal coupling effect in 3D-integrated GaN power modules becomes more pronounced. This leads to a sharp rise in heat accumulation within the system, resulting in a significant temperature increase, ultimately causing degradation in its performance. Currently, international research on the thermal characteristics of GaN power devices primarily focuses on discrete devices. *Lidow A.* studied the effects of temperature on the breakdown voltage and on-resistance of commercial GaN power devices in 2010, revealing that an increase in temperature leads to higher resistance and a lower breakdown voltage [29]. In 2015, Zhou Q. et al. also published research on the effects of temperature on the breakdown voltage and on-resistance of GaN power diodes [30,31]. Higher operating temperatures in GaN power diodes result in greater reverse leakage, lower breakdown voltage, and higher on-resistance. In 2017, Shireen Warnock et al. comprehensively investigated the impact of temperature on the gate dielectric breakdown characteristics of GaN power devices, proposing an incremental breakdown theory to explain why the dielectric breakdown voltage decreases with increasing temperature [32]. In 2018, Shi. et al. discovered that GaN power devices subjected to repetitive pulse overcurrent experience performance degradation due to high temperature gradients generated by high electric fields and large currents, leading to the formation of defects [33]. Currently, there is no research available on the mutual thermal coupling between multiple GaN power devices within 3D-integrated GaN power modules. The operating characteristics of any GaN power chip in such a module are influenced by the thermal coupling effects from the other devices. Therefore, it is crucial to study the mechanism of mutual thermal coupling between devices in 3D-integrated GaN power modules to provide support for enhancing the application reliability of these modules.

This work focuses on the analysis of characteristics in 3D-integrated GaN power modules under multi-heat source coupling. It investigates the impact of the heat generated by a single-layer chip in a 3D-integrated GaN power module and the thermal coupling effect between different chips on the characteristics of the other GaN power chips. The structure of this paper is as follows: the Section 2 introduces the experimental setup, the Section 3 presents the experimental results and discussion, and the conclusions of this work are provided in the Section 4.

## 2. Sample Structure and Experimental Setup

**Figure 1:**
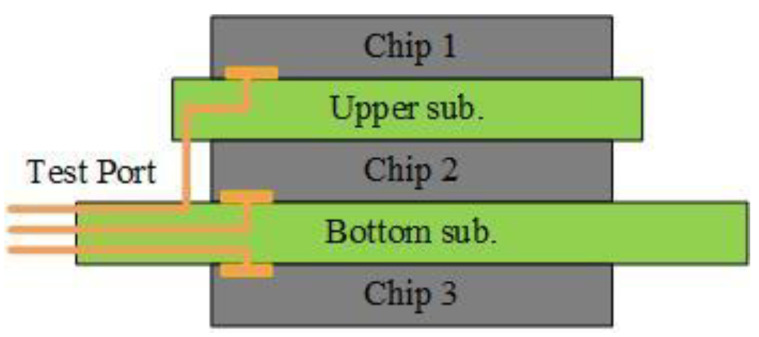
Schematic Diagram of the 3D-integrated GaN Power Module.

**Figure 2:**
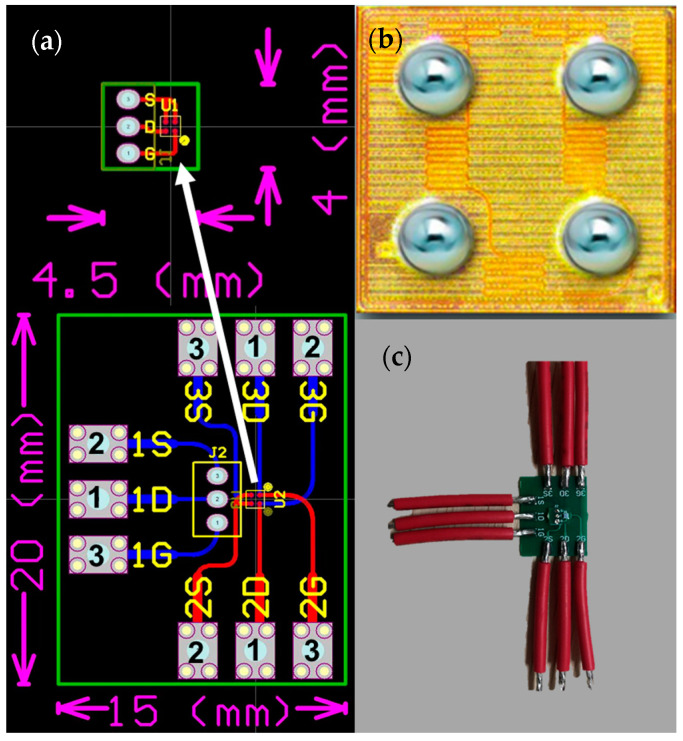
Electrical Connections and Physical Diagram of the 3D-integrated GaN Power Module: (**a**) Electrical Connections; (**b**) Chip Electrodes; (**c**) Physical Diagram.

To reduce experimental costs, this work utilizes the *E*-mode GaN power transistor (EPC2036) to construct a 3D-integrated GaN power module. Three EPC2036 chips are installed on two layers of PCB substrates with a thickness of 0.4 mm. The upper substrate measures 4.5 mm × 4 mm, while the lower substrate measures 20 mm × 15 mm. The chips are named sequentially from top to bottom as Chip 1, Chip 2, and Chip 3. Chip 1 is connected to the lower substrate for a unified power supply via pin headers, while Chips 2 and 3 are fixed to the front and back of the lower substrate. The stacking structure of the three chips is shown in Figure 1. To facilitate testing, electrical pins for the gate (G), drain (D), and source (S) of each chip are led out from three directions on the lower substrate. The physical sample of the 3D-integrated GaN power module is shown in Figure 2.

**Figure 3:**
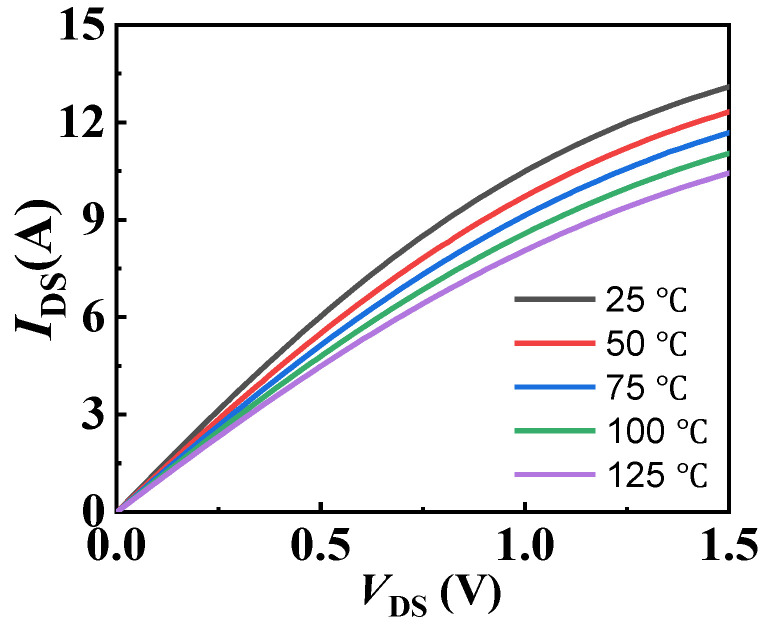
The temperature-dependent output characteristics of EPC2036.

Figure 3 exhibits the temperature-dependent output characteristics of EPC2036. Although GaN materials can withstand high temperatures, devices based on GaN materials exhibit significant changes in their characteristics after temperature fluctuations, even if it is only a few tens of degrees. The operation of the GaN power chip in a 3D-integrated GaN power module will generate high temperatures, subsequently leading to variations in the operating states of the other GaN power chip. The analysis in this work will provide insights into the reliability of the 3D-integrated GaN power module in multi-thermal source coupling environments. This experiment selected three 3D-integrated GaN power module samples (labeled as Sample #1, Sample #2, and Sample #3) to study the thermal coupling effects on the characteristics of the GaN power chips in the first, second, and third layers. Before starting the experiment, the initial parameter values of the GaN power chips (in the first layer of Sample #1, the second layer of Sample #2, and the third layer of Sample #3) were tested separately by B1505A (From Keysight Technology Co., Ltd., Santa Rosa, CA, USA). For the transfer characteristics curves, *V*_GS_ increases to 3.5 V from 1 V with a step of 0.02 V and *V*_DS_ of 3 V; For the output characteristics curves, *V*_DS_ increases to 1.3 V from 0 V with a step of 0.01 V and *V*_GS_ of 4 V; For the drain leakage curves, *V*_DS_ is increased to 100 V from 0 V with a step of 0.15 V and *V*_GS_ of 0 V; For the gate delay curve, *V*_DS_ is set to 0.4 V, and *V*_DS_ switches instantly from 0 V to *V*_GS_ = 3 V after 100 s, and the change curve of output current is recorded. After testing the initial data of all samples, we applied *V*_GS_ = 3 V and *V*_DS_ = 1 V to one or two power chips within the 3D-integrated GaN power module and maintained this condition for 60 s (By N6976A from Keysight Technology Co., Ltd, Santa Rosa, CA, USA). Then, we measured again the electrical performance of the target layer chips. In this work, to avoid temperature changes in the chip itself during parameter testing [34], all high current tests are conducted in fast testing mode, which can be completed in a shorter time. Each layer of chips underwent three different coupling environment control tests, as detailed in Table 1. For example, in the coupling relationship of Sample #1, “2-to-1” indicates that the second-layer chip is in the conducting state, and “2/3-to-1” indicates that both the second- and third-layer chips are in the conducting state. The meanings of other coupling relationships in the table are similar. Each sample was tested for its different layer chips under various thermal coupling environments, and the parameter values were compared with the initial values to determine the drift amount.

## 3. Results and Discussion

**Figure 4:**
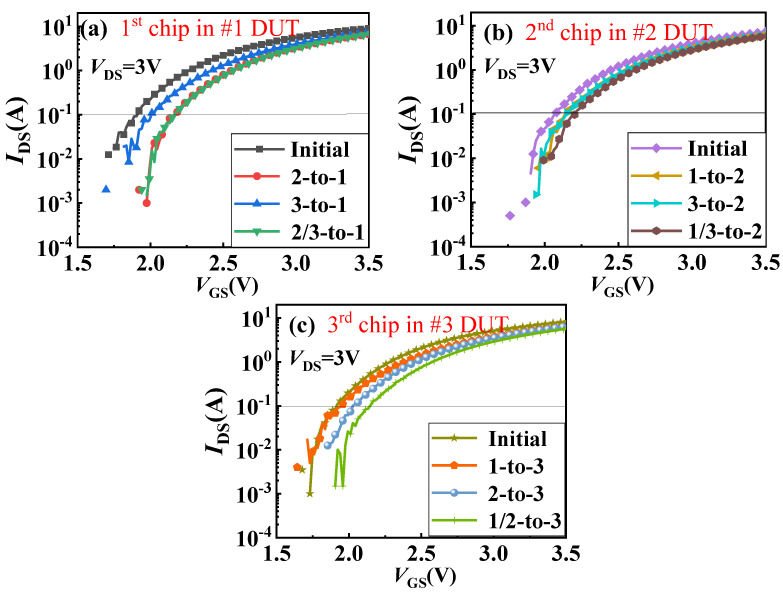
Transfer curves of different layer chips in 3D-integrated GaN power modules under thermal coupling effect: (**a**) transfer curves of first-layer chip of Sample #1 under influence of 2-to-1, 3-to-1, and 2/3-to-1 modes, (**b**) transfer curves of second-layer chip of Sample #2 under influence of 1-to-2, 3-to-2, and 1/3-to-2 modes, (**c**) shows transfer curves of third-layer chip of Sample #3 under influence of 1-to-3, 2-to-3, and 1/2-to-3 modes.

**Figure 5:**
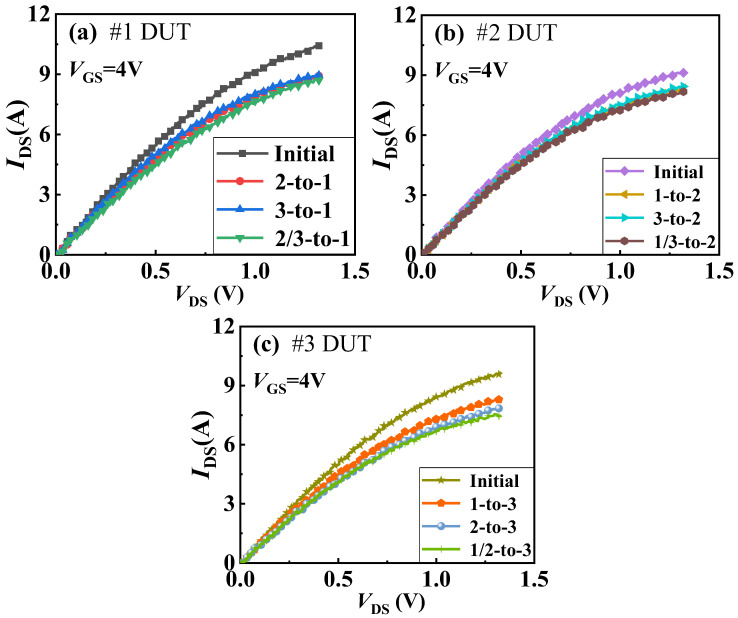
Output curves of different layer chips in 3D-integrated GaN power modules under thermal coupling effect: (**a**) output curves of first-layer chip of Sample #1 under influence of 2-to-1, 3-to-1, and 2/3-to-1 modes, (**b**) output curves of second-layer chip of Sample #2 under influence of 1-to-2, 3-to-2, and 1/3-to-2 modes, (**c**) shows output curves of third-layer chip of Sample #3 under influence of 1-to-3, 2-to-3, and 1/2-to-3 modes.

Figure 4 and Figure 5 present the impact of the thermal coupling effect on the transfer and output characteristics of the GaN power chips in a 3D-integrated GaN power module. Specifically, Part (a) shows the changes in transfer and output characteristics curves of the first-layer chip in Sample #1 before and after other layers’ chips are operated. Part (b) illustrates similar changes for the second-layer chip in Sample #2 and Part (c) for the third-layer chip in Sample #3. Due to this, the process of GaN power devices is currently not particularly mature and there are certain differences in the initial performance of the devices. From these figures, it is evident that regardless of which layer the chip is in, when subjected to heat coupling from other layers, its threshold voltage (defined at drain current of 100 mA (*I*_DS_ = 100 mA)) shifts positively, and the on-current decreases. This is due to the temperature rise caused by heat from other layers, which accelerates the random movement of free electrons, leading to a decrease in carrier mobility in the channel.

**Figure 6:**
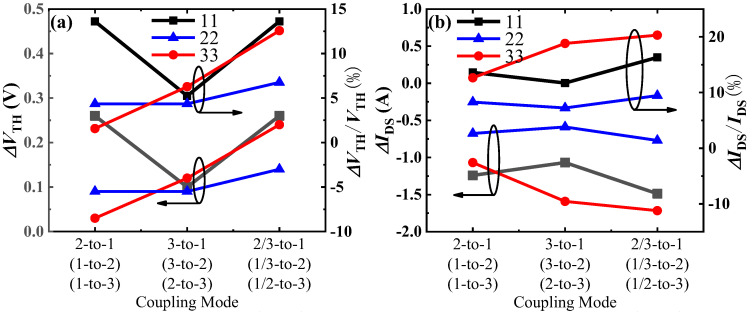
(**a**) Threshold voltage shift and (**b**) on-current shift under different thermal coupling modes.

**Figure 7:**
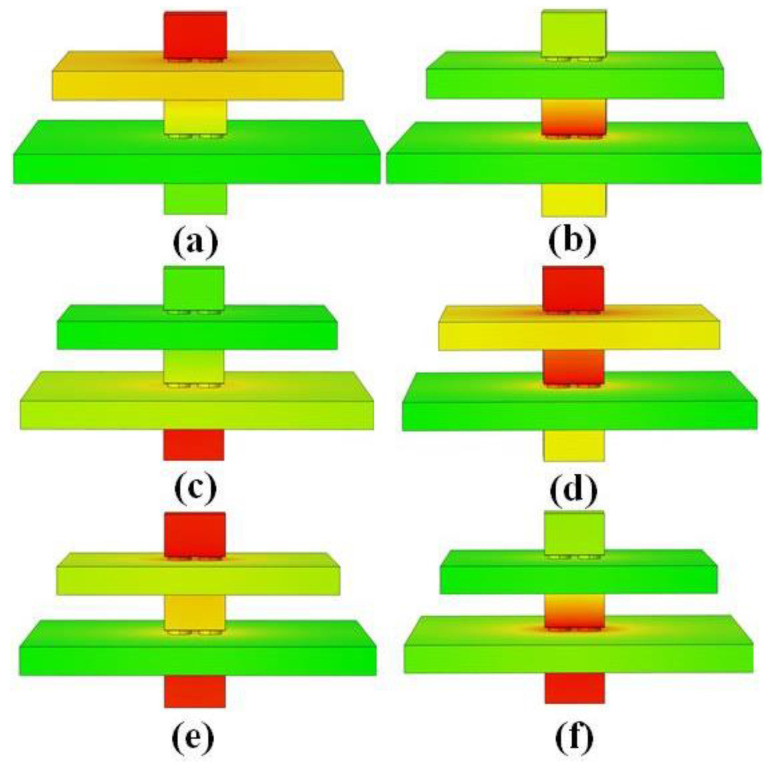
Multi-Heat Source Coupling Effects in 3D-Integrated GaN Power Modules: (**a**) First-layer chip working alone; (**b**) Second-layer chip working alone; (**c**) Third-layer chip working alone; (**d**) First-layer and second-layer chips working simultaneously; (**e**) First-layer and third-layer chips working simultaneously; (**f**) Second-layer and third-layer chips working simultaneously.

To analyze the impact of different thermal coupling modes on the characteristics of the GaN power chips, we have statistically analyzed the changes in threshold voltage and on-current (@*V*_GS_ = 4 V, *V*_DS_ = 1 V), as shown in Figure 6. Firstly, we will analyze the changes in the threshold voltage and on-current of the first-layer chip in Sample #1. According to the data shown in Figure 6, the change rates of measured threshold voltage and on-current are 13.61% and 16.32%, respectively, under the 2/3-to-1 coupling mode; 13.61% and 13.61%, respectively, under the 2-to-1 coupling mode; and 5.24% and 11.71%, respectively, under the 3-to-1 coupling mode. Although there is no change in the transfer characteristics of #1 for 2-to-1 (red in Figure 4) and 2/3-to-1 (green curves in Figure 4), there is a small change in on-current of #1 for 2-to-1 (red curve in Figure 5) and 2/3-to-1 (green curve in Figure 5). This indicates that the impact of other layers’ chips on its parameters follows the order: the simultaneous operation of the second- and third-layer chips has a greater impact on the first-layer chip than the impact of the second-layer chip alone, and the impact of the third-layer chip alone is the least. It suggests that the closer the distance between two chips, the more susceptible they are to the thermal field generated during operation. Moreover, when the second- and third-layer chips operate simultaneously, the superposition of heat will exacerbate the parameter degradation of the first-layer chip, as shown in Figure 7.

For the 3D-integrated GaN power module, when only one chip is in operation, the thermal field generated by it will cause the temperature rise of the adjacent chips. For example, when the first-layer chip is in operation, the temperature of the second-layer chip will rise, while the temperature rise of the third-layer chip is relatively small. When the second-layer chip is in operation, the temperatures of both the first-layer and the third-layer chips will rise. When the third-layer chip is in operation, the temperature of the second-layer chip will rise, while the temperature rise of the first-layer chip is relatively small. In addition, when two layers of chips are in operation simultaneously, the chip’s temperature rise is greater than that when only one chip is in operation. In addition, this phenomenon can also be explained by thermal resistance theory. Due to the distance between the third-layer chip and the first-layer chip, the thermal resistance between the third-layer chip and the first-layer chip is relatively high. Therefore, the heat generated during the operation of the third-layer chip has a relatively small impact on the first-layer chip. Similarly, the heat generated during the operation of the first-layer chip has a relatively small impact on the third-layer chip. For the case where the third-layer and second-layer chips work simultaneously, due to the high temperature of both the second-layer and third-layer chips, the heat of the second-layer chip tends to transfer to the first-layer chip, resulting in more changes in the performance of the first-layer chip. Therefore, it is necessary to avoid multiple layers of chips working simultaneously and reduce their impact.

The change rates of the threshold voltage and on-current of the second-layer chip in Sample #2 are as follows: 6.76% and 9.45%, respectively, under the 1/3-to-2 coupling mode; 4.35% and 8.29%, respectively, under the 1-to-2 coupling mode; and 4.35% and 7.23%, respectively, under the 3-to-2 coupling mode. This indicates that the impact of other layers’ chips on its parameters, from greatest to least, is as follows: the simultaneous operation of the first- and third-layer chips has a greater impact on the second-layer chip than the impact of the first-layer chip alone, and the impact of the third-layer chip alone is the least. Since the first-layer chip and the third-layer chip are located above and below the second-layer chip, respectively, the heat transfer comes from two directions, resulting in a greater coupling effect. The heat coupling from the third-layer chip to the second-layer chip is less than that from the first-layer chip, possibly due to the third-layer chip being situated on a larger substrate at the bottom. According to the theory of heat transfer in solids, heat always flows towards the direction of lower thermal resistance. Therefore, most of the heat generated by the third-layer chip is transmitted into the substrate, reducing its impact on the second-layer chip, as shown in Figure 7.

The degradation rates of the threshold voltage and on-current of the third-layer chip in Sample #3 are as follows: 12.57% and 20.31%, respectively, under the 1/2-to-3 coupling mode; 6.28% and 18.83%, respectively, under the 2-to-3 coupling mode; and 1.7% and 12.65%, respectively, under the 1-to-3 coupling mode. This indicates that the impact of other layers’ chips on its parameters, from greatest to least, is as follows: the simultaneous operation of the first- and second-layer chips has a greater impact on the third-layer chip than the impact of the second-layer chip alone, and the impact of the first-layer chip alone is the least. A comprehensive analysis of the above data reveals that for the third-layer chip in Sample #3, the drift in its parameters is greater than that of the first-layer chip in Sample #1 and the second-layer chip in Sample #2. The main reason for this is likely that the third-layer chip is close to the bottom substrate, but most of the heat generated by the first- and second-layer chips above it is transmitted downward, exacerbating heat accumulation and significantly increasing the degradation of the third-layer chip. Therefore, for this type of three-layer stacked GaN chip, it is advisable to avoid simultaneous operation of the upper two layers of chips to reduce the impact on the characteristics of the lower-layer chip. Additionally, during chip stacking, high-power chips should be placed closer to the bottom substrate to reduce heat accumulation.

**Figure 8:**
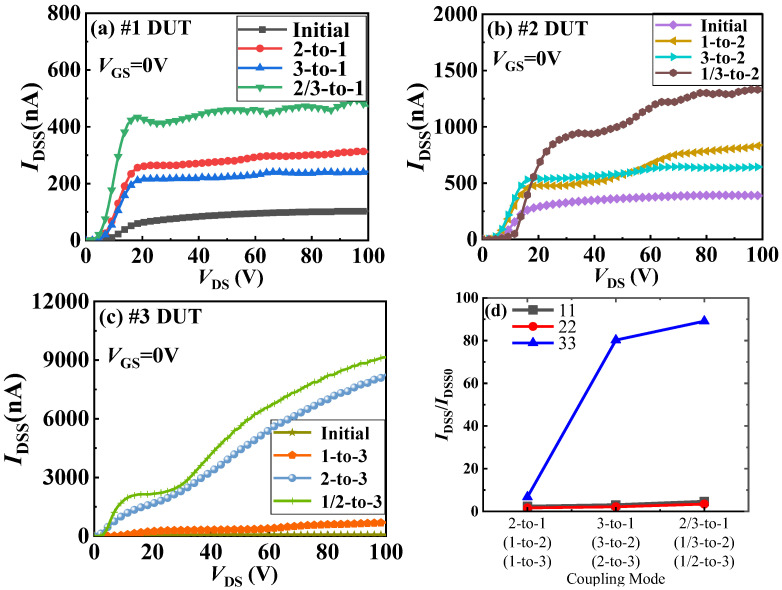
*I*_DSS_ curves of different layers in 3D-integrated GaN power modules under thermal coupling: (**a**) first-layer chip in Sample #1, (**b**) second-layer chip in Sample #2, (**c**) third-layer chip in Sample #3, (**d**) change in *I*_DSS_@*V*_DS_ = 100 V.

Leakage current in power devices is one of the factors causing system losses, which not only reduces the output efficiency of the devices but also disrupts the normal operation of the entire system. Currently, the leakage current in GaN power devices mainly includes the off-state current (*I*_DSS_) between the drain and source channels and the gate leakage current (*I*_GS_). Next, we will analyze the impact of the heat generated by a specific layer of chips in a 3D-integrated GaN power module on *I*_DSS_ of other layers of chips. Figure 8 shows the trend of *I*_DSS_ in the 3D-integrated GaN power module before and after thermal coupling. Figure 8a–c represents the off-state current of the first, second, and third layers of chips, respectively, under different coupling conditions. It can be seen from the figure that the thermal coupling generated by the operation of any one chip will always cause an increase in *I*_DSS_ of the other chips. This is because when a layer of chips is affected by the heat generated by other layer chips, it accelerates the disorderly movement of carriers within it, making it easier for electrons to gain energy and break free from their original restraints, resulting in a larger leakage current. It can also be seen from the figure that the thermal coupling effect has the least impact on *I*_DSS_ of the top layer of chips and the greatest impact on *I*_DSS_ of the bottom layer of chips. As shown in Figure 8a, *I*_DSS_ of the first-layer chip in Sample #1, when the second- and third-layer chips are operating simultaneously, is approximately 500 nA, increasing by about four times (as shown in Figure 8d). In contrast, *I*_DSS_ of the third-layer chip in Sample #3, when the first- and second-layer chips are operating simultaneously, is approximately 9000 nA (as shown in Figure 8c), increasing by nearly a hundred times (as shown in Figure 8d). It also can be seen that some *I*_DSS_ data is oscillated. The oscillated trend of leakage current at high temperatures is generally determined by the distribution and density of traps within the device and does not exhibit periodicity. There are lattice defects and impurities in the bulk material of GaN power devices. These lattice defects or impurities can act as electron traps or hole traps. Some traps may capture electrons or holes at room temperature. As the temperature rises, these traps will release electrons or holes. Under the action of a high voltage at the drain electrode, these traps will continuously capture electrons or holes and raise the barrier height, which in turn causes the oscillated leakage current at high temperatures.

**Figure 9:**
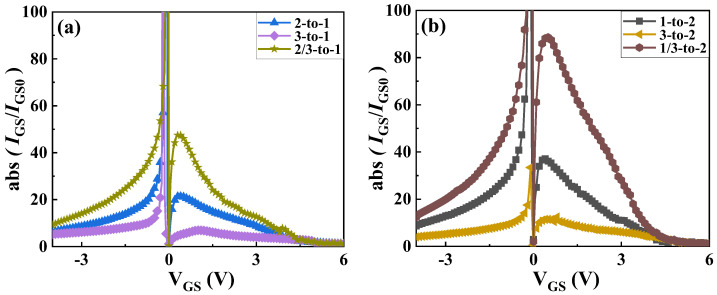
Variation in gate leakage current of different layers in 3D-integrated GaN power modules. (**a**) first-layer chip in Sample #1, (**b**) second-layer chip in Sample #2.

Because gate leakage current is highly sensitive to temperature and difficult to recover in the short term after heating, only the first six coupling relationships are analyzed in order to reduce interference, and six samples are selected for testing, respectively. The ratio of the change before and after is calculated. During the test, the source and drain of the device are shorted, and *V*_GS_ is scanned from −4 V to 6 V. Figure 9 shows the variation of gate leakage current of chips in different layers after different thermal coupling effects. It can be seen from the figure that the thermal coupling generated by any one chip in operation will always lead to an increase in the gate leakage current of other layers of chips. The effect of heat generated by two chips operating simultaneously is greater than that of a single chip operating alone. For the first-layer chip of Sample #1, under the influence of the second- and third-layer chips operating simultaneously, the gate leakage current increases by about eight times (at *V*_GS_ = −4 V), and under the influence of the second-layer chip operating alone, it increases by about four times. For the second-layer chip of Sample #2, under the influence of the first- and third-layer chips operating simultaneously, the gate leakage current increases by about 12 times (at *V*_GS_ = −4 V), and under the influence of the third-layer chip operating alone, it increases by about three times.

**Figure 10:**
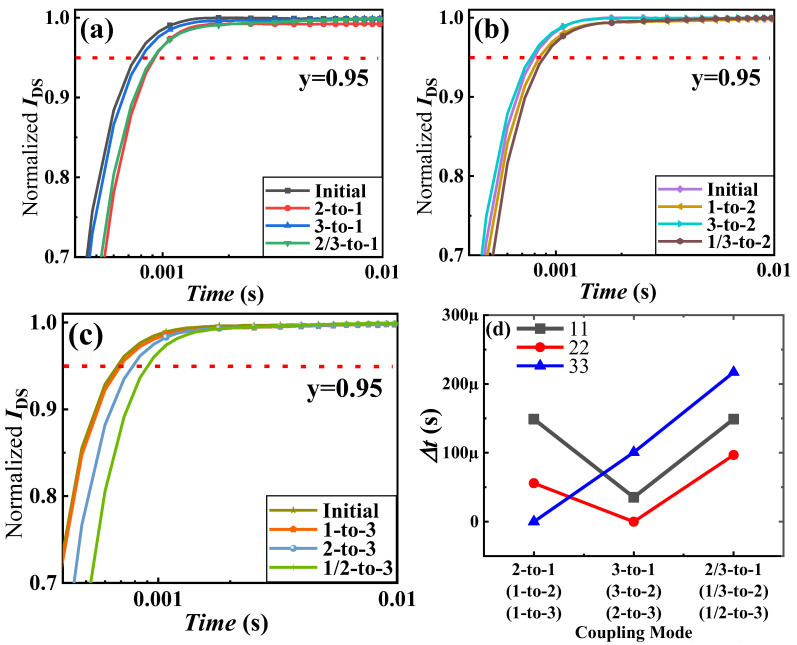
Gate delay in 3D-integrated GaN power modules under different coupling modes: (**a**) first-layer chip of Sample #1, (**b**) second-layer chip of Sample #2, (**c**) third-layer chip of Sample #3, (**d**) variation in gate delay.

Next, the impact of thermal coupling effects on the gate delay of devices in 3D-integrated GaN power modules will be analyzed. Figure 10a–c shows the output current variation curves for the first-, second-, and third-layer chips of Samples #1, #2, and #3, respectively, under the condition where *V*_DS_ = 0.4 V and *V*_GS_ is switched instantaneously to 3 V after being held at 0 V for 100 s. To facilitate the analysis, the channel current values on the y-axis are normalized, representing the percentage of the maximum current. To assess the effect of thermal coupling on the gate delay, the intercepts at y = 0.95 in Figure 10a–c are taken. In Figure 10a, for the first-layer chip of Sample #1, the time required for the output current to reach 95% of the maximum current after the gate turns on is 2-to-1 mode > 2/3-to-1 mode > 3-to-1 mode > initial value. This indicates that for the first-layer chip of Sample #1, the impact on the turn-on speed is similar when the second layer or both the second and third layers are operating, and this impact is greater than when only the third layer is operating. Similarly, in Figure 10b, the time required for the turn-on current to reach 95% of the maximum current is 1/3-to-2 mode >1-to-2 mode >3-to-2 mode = initial value. This means that for the second-layer chip of Sample #2, the impact on the turn-on speed is significant when the first layer or both the first and third layers are operating, and the impact is smaller when only the third layer is operating. In Figure 10c, the time required for the turn-on current to reach 95% of the maximum current is 1/2-to-3 mode >2-to-3 mode >1-to-3 mode = initial value. This indicates that for the third-layer chip of Sample #3, the impact on the turn-on speed is significant when the second layer or both the first and second layers are operating, and the impact is smaller when only the first layer is operating. The increased device’s response time at high temperatures is related to the trapping-related effect within the device. As the temperature rises, the traps in the bulk material of GaN power devices will release electrons or holes. During the turn-on process of devices, these traps will capture electrons or holes, subsequently increasing the device’s response time.

## 4. Conclusions

This study employs an E-mode GaN power transistor to develop a 3D-integrated GaN power module and investigates the impact of the heat generated by a single chip layer and the thermal coupling effect between different chips on the characteristics of the other GaN power chips. The results demonstrate that thermal coupling in the 3D-integrated GaN power module induces a shift in the device’s threshold voltage toward higher values, reducing the device’s response speed and current output capability. Furthermore, it also increases the leakage current of the GaN power chips. In severe cases, the threshold voltage drift can reach up to 0.26 V, the device’s response time can increase by as much as 217 μs, the on-state current can decrease by up to 1.7 A, and the off-state leakage current can increase by more than 80 times. The influence of thermal coupling on chip performance is closely related to the direction of heat flow and the distance between chips. The closer the chips are to each other, the stronger the thermal coupling effect. Chips located near the bottom substrate experience a more significant impact from thermal coupling, while those at the top of the module are less affected. Additionally, the thermal coupling effect of two chips is typically greater than that of a single chip. Therefore, for three-dimensional integrated GaN power modules, it is necessary to avoid multiple layers of chips working simultaneously and reduce their impact. When designing three-dimensional integrated GaN power modules, the chips with higher power should be placed on the underlying substrate to reduce their impact.

## Figures and Tables

**Table 1:** Test Groups for 3D-integrated GaN power module.

Sample No.	Chip No.	Target Chip	Coupling Mode
#1	11	The first-layer chip of Sample #1	2-to-1: The second-layer chip is in the conducting state, and its influence on the first-layer chip.
	12		3-to-1: The third-layer chip is in the conducting state, and its influence on the first-layer chip.
	13		2/3-to-1: Both the second- and third-layer chips are in the conducting state, and its influence on the first-layer chip.
#2	21	The second-layer chip of Sample #2	1-to-2: The first-layer chip is in the conducting state, and its influence on the second-layer chip.
	22		3-to-2: The third-layer chip is in the conducting state, and its influence on the second-layer chip.
	23		1/3-to-2: Both the first- and third-layer chips are in the conducting state, and their influence on the second-layer chip.
#3	31	The third-layer chip of Sample #3	1-to-3: The first-layer chip is in the conducting state, and its influence on the third-layer chip.
	32		2-to-3: The second-layer chip is in the conducting state, and its influence on the third-layer chip.
	33		1/2-to-3: Both the first- and second-layer chips are in the conducting state, and their influence on the third-layer chip.

## Data Availability

The original contributions presented in this study are included in the article. Further inquiries can be directed to the corresponding authors.
